# *Moraxella catarrhalis* NucM is an entry nuclease involved in extracellular DNA and RNA degradation, cell competence and biofilm scaffolding

**DOI:** 10.1038/s41598-019-39374-0

**Published:** 2019-02-22

**Authors:** Aimee Tan, Wing-Sze Li, Anthony D. Verderosa, Luke V. Blakeway, Tsitsi D. Mubaiwa, Makrina Totsika, Kate L. Seib

**Affiliations:** 1 Institute for Glycomics, Griffith University, Gold Coast, Queensland 4215 Australia; 2 Institute of Health and Biomedical Innovation, School of Biomedical Sciences, Queensland University of Technology, Brisbane, Queensland 4006 Australia

## Abstract

*Moraxella catarrhalis* is a host-adapted bacterial pathogen that causes otitis media and exacerbations of chronic obstructive pulmonary disease. This study characterises the conserved *M*. *catarrhalis* extracellular nuclease, a member of the ββα metal finger family of nucleases, that we have named NucM. NucM shares conserved sequence motifs from the ββα nuclease family, including the DRGH catalytic core and Mg^2+^ co-ordination site, but otherwise shares little primary sequence identity with other family members, such as the *Serratia* Nuc and pneumococcal EndA nucleases. NucM is secreted from the cell and digests linear and circular nucleic acid. However, it appears that a proportion of NucM is also associated with the cell membrane and acts as an entry nuclease, facilitating transformation of *M*. *catarrhalis* cells. This is the first example of a ββα nuclease in a Gram negative bacteria that acts as an entry nuclease. In addition to its role in competence, NucM affects cell aggregation and biofilm formation by *M*. *catarrhalis*, with Δ*nucM* mutants having increased biofilm biomass. NucM is likely to increase the ability of cells to survive and persist *in vivo*, increasing the virulence of *M*. *catarrhalis* and potentially affecting the behaviour of other pathogens that co-colonise the otorhinolaryngological niche.

## Introduction

*Moraxella catarrhalis* is a Gram-negative, human-restricted bacterial species that colonises the upper and lower respiratory tracts. *M*. *catarrhalis* can be carried asymptomatically (known as carriage), but can also cause diseases such as otitis media (OM) and exacerbations of chronic obstructive pulmonary disease (COPD). OM is primarily a childhood disease, and in addition to the immediate consequences of pain and inflammation, severe infection can lead to complications such as hearing loss and learning deficiencies^1–3^. *M*. *catarrhalis* is currently the third most common bacterial pathogen associated with OM^4^. In contrast, COPD is a disease of the elderly whereby repeated exacerbations lead to loss of lung function and eventually death, and *M*. *catarrhalis* is the second leading cause of bacterial induced exacerbations of COPD^5,6^. However, several studies suggest that *M*. *catarrhalis* is under-reported in disease cases^4,5^, and the increasing use of molecular methods for pathogen detection may see the burden of disease attributed to *M*. *catarrhalis* rise. A shift in the aetiology of OM and COPD may also occur, with an increased burden of disease caused by *M*. *catarrhalis*, due to vaccination programs directed against the other major otopathogens, *Streptococcus pneumoniae* and *Haemophilus influenzae*^5,7^.

One notable and distinguishing feature of *M*. *catarrhalis* strains is a high level of nuclease activity external to the cell, which can be used to distinguish them from other *Moraxella* species on agar^8^. Research on *M*. *catarrhalis* has noted nuclease activity for over 30 years^9^, yet to date, little work has been done to investigate its basis. Extracellular nucleases have been characterised in a number of pathogenic bacterial species, including the other major otopathogens *S*. *pneumoniae*^10–12^ and *H*. *influenzae*^13^, and other bacterial pathogens such as *Vibrio cholerae*^14^, *Streptococcus pyogenes*^15^, *Staphylococcus aureus*^16,17^ and *Neisseria gonorrhoeae*^18^.

Extracellular nucleases have diverse roles. Early studies identified nucleases in *B*. *subtilis* and *S*. *pneumoniae* as essential components for natural competence, the uptake of DNA from the environment into the cell^12,19^. These nucleases are part of the Nuc superfamily (also called DNA/RNA nonspecific endonucleases). The function of the nuclease in competence is best described with the pneumococcal EndA, which complexes with competence machinery and generates a single stranded DNA molecule that can be transported across the cell membrane^20^. Competence has been hypothesised to contribute to cellular fitness in a number of ways, including nutrition, genome repair and evolution^20^.

The contribution of extracellular nucleases to cell growth and survival strategies, such as in biofilm formation, has also been described. Biofilms are multi-cellular bacterial communities that enhance bacterial adhesion, persistence, and resistance to antimicrobials, and are problematic in both environmental (such as biofouling in aquatic environments)^21^ and clinical settings (such as on medical instruments, implants and in sites of pathology, such as in cystic fibrosis lungs, the oral cavity and middle ear^22–24^). A critical component of the biofilm matrix is extracellular DNA (eDNA), which facilitates cell adhesion and interaction, and contributes to the integrity and maintenance of biofilm structure^25,26^. The removal of eDNA (e.g., by application of nucleases such as DNAseI) has been shown to disrupt biofilm formation and maintenance in a number of bacterial species^25,27^. Extracellular nucleases are also used to target biofilm eDNA in cases of cystic fibrosis, and have been considered in other conditions to target biofilm-forming species^28,29^. The effect of nuclease production on biofilm formation in bacterial species that produce their own extracellular nucleases has been examined in a range of species, including *V*. *cholerae*^14^, *H*. *influenzae*^13^, *S*. *aureus*^16^ and *N*. *gonorrhoeae*^30^. In these cases, disruption of the nuclease gene has correlated with increased biofilm mass and accumulation of eDNA to levels exceeding the parental strain. Other studies have examined the ability of nucleases to facilitate escape from neutrophil extracellular traps (NETs), which use secreted DNA to immobilise bacteria and deliver antimicrobial components^10,15,17,18,31–33^; to enable use of DNA as a nutritional source^34^; and in some cases (such as Rv0888 from *M*. *tuberculosis*) to facilitate acquisition of other nutrients^35,36^.

This study characterises the gene encoding the extracellular nuclease of *M*. *catarrhalis*, which we have termed *nucM*, and its conservation and distribution. The cellular location and degradation activity of NucM are also investigated by deletion of the *nucM* reading frame. Finally, we characterise the effect of NucM on *M*. *catarrhalis* growth, competence and biofilm formation.

## Results

### *M*. *catarrhalis* encodes an extracellular nuclease

**Figure 1:**
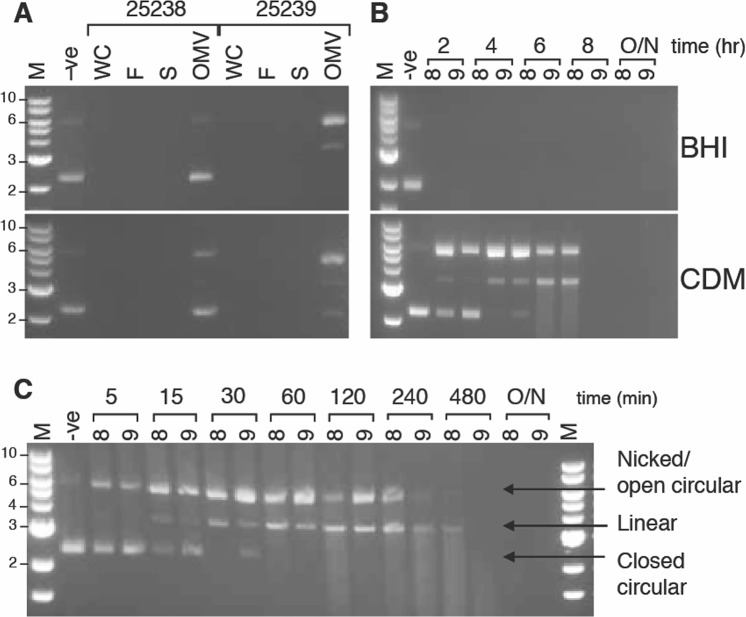
Extracellular nuclease activity of *M*. *catarrhalis*. (**A**) Nuclease activity in whole cell and secreted fractions of *M*. *catarrhalis* strain 25238 and 25239 cells grown in brain heart infusion media (BHI; top) and chemically defined media (CDM; bottom), co-incubated with plasmid DNA overnight. Fractions are whole cell (WC), cell free filtrate (F), supernatant from ultracentrifugation of OMVs (S) and outer membrane vesicles (OMV). Sizes are given for the marker (M; 1 kb ladder, NEB) in the bottom panel. (**B**) Nuclease activity in BHI (top panel) and CDM (bottom panel) at 2 hour intervals (representing early-, mid-, late-log and stationary phases during aerobic growth) for *M*. *catarrhalis* strains 25238 (8) and 25239 (9), showing shift in plasmid forms from closed circular to nicked and linear forms in CDM isolated filtrate. (**C**) Sequential degradation of plasmid DNA over time. Nuclease activity from cell-free filtrate from overnight aerobic growth of *M*. *catarrhalis* 25238 (8) and 25239 (9) strains in BHI, co-incubated with plasmid DNA for timed intervals of 5, 15, 30, 60, 120, 240 or 480 minutes, or overnight (O/N). Plasmid forms are indicated to the right of the gel, and sizes are given for the marker (1 kb ladder, NEB) to the left. The full-length gels from panels A–C are presented in Supplementary Fig. S3.

Extracellular DNase activity is a common characteristic of *M*. *catarrhalis*, however the localisation and identity of the nuclease is unknown. Therefore, the localisation of nuclease activity of *M*. *catarrhalis* wild type strains 25238 and 25239 was initially investigated by examining cell fractions of bacteria grown to mid log in rich (BHI) and chemically defined media (CDM) broths. As activity was expected to be extracellular, whole cells were centrifuged and resuspended in fresh media, and the supernatant was filtered through 0.22 µm filters to ensure it was cell-free and filtrate was further separated into OMV and secreted protein supernatant fractions by ultracentrifugation. Incubation of these fractions with 100 ng plasmid DNA resulted in DNA degradation by whole cell, cell filtrate and supernatant fractions of *M*. *catarrhalis*. OMVs showed only partial degradation of the plasmid, with more appearing as nicked and linear DNA than intact plasmid (Fig. 1A). This suggests that the majority of the nuclease is secreted into the growth medium, but that there may also be a portion of the nuclease associated with the cell membrane (as evidenced by OMV-mediated DNA degradation).

Analysis of nuclease activity during different stages of growth was investigated by assessing nuclease activity in filtrate samples taken from aerobic growth of parental strains 25238 and 25239 in BHI and CDM at early-, mid- and late-log, and stationary phase. Cells grown in BHI have substantial nuclease activity throughout the entire growth phase. In contrast, full degradation of plasmid DNA is only seen after 6 hours of growth (entering late-log to stationary phase) for cells grown in CDM, suggesting that nuclease expression and/or activity is not sufficient to degrade DNA until later growth phases, or until accumulation of high enough levels, in this media (Fig. 1B). Interestingly, degradation of the plasmid DNA appears to occur in a sequential manner, with the plasmid first being nicked (shift from closed circular form to nicked/relaxed circular), then linearised before degradation occurs. This sequential degradation is also seen when the effect of incubation time on nuclease activity is examined (i.e. the transition from closed circular to nicked/relaxed circular to linear is seen as incubation time increases, Fig. 1C).

### NucM is non-essential to *M*. *catarrhalis*, and degrades nucleic acids

**Figure 2:**
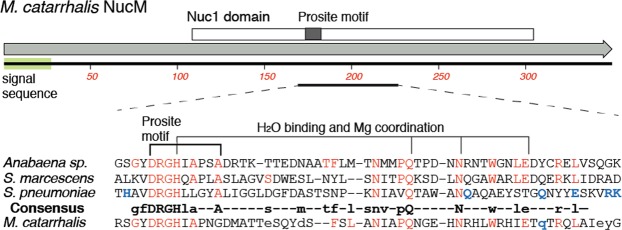
Conserved DRGH nuclease motif. Partial protein sequence alignment of the *M*. *catarrhalis* NucM consensus to the *Serratia* nuclease and pneumococcal End A. Alignment of the central region of the NucM protein consensus from *M*. *catarrhalis* (with non-conserved residues shown in lowercase) to the *Serratia marcescens* nuclease, *S*. *pneumoniae* EndA and consensus sequence from multispecies alignments as seen in^42^. Matches to the consensus sequence are shown in red, with additional residues important for the mechanism of EndA shown in blue^45^.

Candidates that might encode the extracellular nuclease were identified in sequenced *M*. *catarrhalis* genome data^37,38^. Analysis of one candidate (the MC25239_RS07280 locus in strain 25239, annotated as a non-specific endonuclease; locus tag MCR_RS06910 in BBH18). This open reading frame is 1050 nucleotides in length and encodes a 349 amino acid, 39.08 kDa protein with a NUC1 domain (COG1864^39^), which is part of the Endonuclease_Non Specific family (Pfam PF01223; Prosite PS01070). Further secondary and tertiary structure modelling indicate the protein is a His-Me finger endonuclease^40^, that PSORTB analysis indicates has extracellular localisation, and SignalP predicted the presence of a signal peptide with cleavage site between pos. 27 and 28. This group is prototypically represented by the *Serratia* Nuc endonuclease^41,42^, and includes nucleases such as EndA from *S*. *pneumoniae*^12^ and nuclease A from *Anabaena* spp^43^. The *M*. *catarrhalis* nuclease, henceforth to be termed NucM, contains the conserved DRGH motif (residues 174–177 in *M*. *catarrhalis* NucM; Fig. 2) that typifies this family of nucleases. It also contains amino acids in positions similar to conserved residues in the *Serratia* nuclease^41,42^ and pneumococcal EndA^44,45^, that are needed for enzyme function – such as in the catalytic and Mg^2+^ co-factor binding core (Fig. 2). However, other than these residues, NucM shares little similarity with other DRGH nucleases (16% identity and 28% similarity to the *Serratia* nuclease, and 16% and 31% to the pneumococcal EndA). Within *M*. *catarrhalis*, NucM is highly conserved, with homologues found in all 54 available *M*. *catarrhalis* genome sequences. These homologues all contain the DRGH motif and putative Mg^2+^ binding residues, and have a minimum protein similarity of 95.99% between homologues. More distantly related homologues are also seen in other species in the *Moraxella* genus and amongst the closely related *Psychrobacter* spp (Fig. S1).

**Figure 3:**
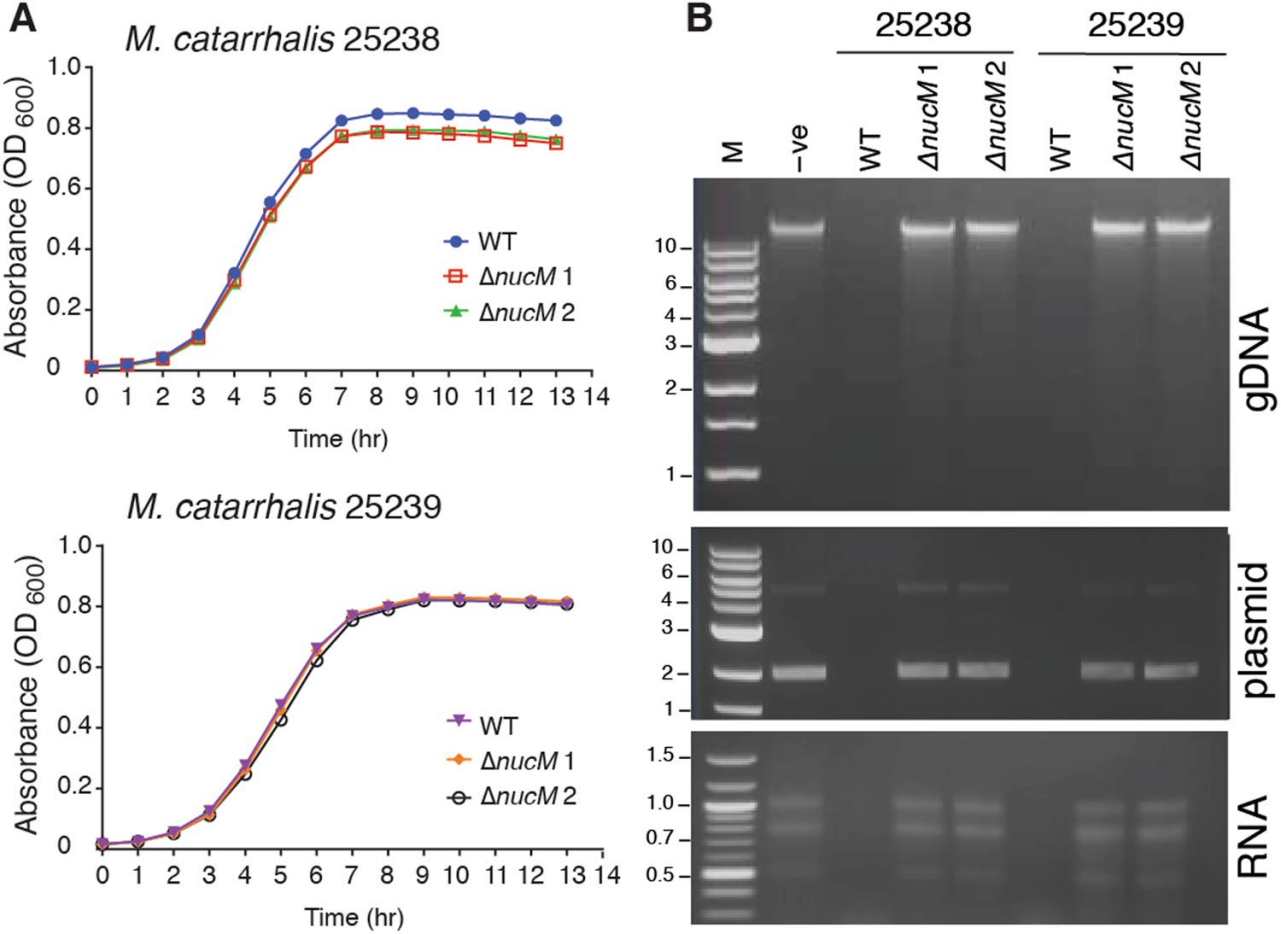
Growth and extracellular nuclease activity of *M*. *catarrhalis* wild type and isogenic *ΔnucM* mutants. (**A**) Growth curve of *M*. *catarrhalis* 25238 and 25239 strains, grown overnight in BHI at 37 °C with aeration, using 0.5 mL aliquots in a 48-well plate format. Assays were carried out in triplicate and OD_600_ values were measured in a Tecan Infinite 200 Pro plate reader, with mean and standard deviation shown. (**B**) Degradation assays of genomic DNA (gDNA), plasmid DNA or RNA incubated with filtered broth without cells (media only, -ve), or grown with wild type (WT) or isogenic *ΔnucM* knock out 25238 and 25239 strains. The full-length gels from panel B are presented in Supplementary Fig. S4.

To analyse the function of NucM in *M*. *catarrhalis*, the *nucM* reading frame was replaced with a kanamycin resistance cassette in *M*. *catarrhalis* strains 25238 and 25239 to produce Δ*nucM* mutant strains. Two approaches to complement these mutations were attempted, using pWW115 (a *M*. *catarrhalis* plasmid that replicates independently in the cell^46^), and pMComCm (an integrative complementation vector designed in this study). The pMComCm vector consists of a chloramphenicol resistance cassette (*cat*), flanked with approximately 400 nucleotides of *M*. *catarrhalis* genome sequence that allows recombination into the *ggt* pseudogene. A multiple cloning site was also integrated downstream of the *cat* gene to facilitate future complementation experiments (Fig. S2). This vector was manipulated and purified in *E*. *coli*, and successfully transforms in *M*. *catarrhalis* strains. However, *nucM* could not be reliably cloned and appears to be deleterious in *E*. *coli* (as is also seen with the pneumococcal *endA* extracellular nuclease^44^), and so a complementation vector could not be constructed. Therefore, all work has been carried out with two independent Δ*nucM* deletion mutants for each strain (Δ*nucM* 1 *and* Δ*nucM* 2). The kanamycin resistance cassette used does not have a functional promoter or terminator, and has previously been used to interrupt genes in an operon with no downstream effects^47^, therefore polar effects from downstream genes are unlikely. These deletions do not affect the aerobic growth of *M*. *catarrhalis* in BHI, with no difference in growth seen between the wild type or the 2 independent Δ*nucM* 1 and Δ*nucM* 2 deletion mutants, indicating that NucM is not essential for growth (Fig. 3A).

To determine if NucM is responsible for degrading nucleic acids, cells were grown to mid-log phase in rich media and filtrates from growth of parental and *ΔnucM* mutants were incubated with DNA (100 ng *M*. *catarrhalis* genomic DNA, or non-specific plasmid DNA) or RNA (200 ng *M*. *catarrhalis* total RNA) at 37 °C overnight. Filtrate from parental strains 25238 and 25239 degraded nucleic acid, but the filtrates from *ΔnucM* mutants did not and were comparable to media-only controls (Fig. 3B). These data indicate that deletion of the *nucM* open reading frame reduces the ability of *M*. *catarrhalis* to digest nucleic acids extracellularly, and that NucM is the enzyme responsible for extracellular nuclease activity.

### NucM is needed for *M*. *catarrhalis* competence

Data from degradation assays suggested that DNA degradation occurs in a processed manner, and that a small amount of the nuclease was associated with cell membranes. This would be consistent with a role in DNA uptake and transformation, as has been seen with the pneumococcal EndA nuclease^12^. To examine the role of NucM in competence, the transformation efficiency of parental and *ΔnucM* mutant strains was determined. For this work, three sources of DNA were used: pWW115, a plasmid with a *M*. *catarrhalis* origin that replicates independently in *M*. *catarrhalis*^46^; pMComCm, a complementation vector that integrates into the genome (described above); and gDNA isolated from 25238 and 25239 strains transformed with the integrative pMComCm vector (termed 25238 MComCm and 25239 MComCm, respectively).

Transformation of cells with the *M*. *catarrhalis* plasmid pWW115 and the integrative plasmid pMComCm demonstrated that *ΔnucM* mutant strains did not have detectable levels of competence (>3–5 log reduction relative to the wild type strains, Table 1). *M*. *catarrhalis* parental strains 25238 and 25239 were transformed with chromosomal DNA at ~1–2 log higher frequency than seen for plasmid DNA. Some transformants were also obtained for *ΔnucM* mutant strains, however these were also at much lower frequencies than the parental strains (≥3-log reduction, Table 1). Collectively, these data indicate that *nucM* is important for the natural competence of *M*. *catarrhalis*.

**Table 1:** Transformation efficiency of wild type and isogenic *ΔnucM* mutants.

DNA source	Strain	WT1	*ΔnucM* 1^a^	*ΔnucM* 2^a^
pWW115 (native plasmid)	25328	8.74 ± 6.34 × 10^−5^	0	0
	25239	3.49 ± 0.91 × 10^−4^	0	0
pMComCm (non-native plasmid)	25328	1.31 ± 0.26 × 10^−5^	0	0
	25239	1.82 ± 0.93 × 10^−3^	0	0
gDNA (native genomic DNA)	25328	2.16 ± 0.16 × 10^−3^	3.52 ± 3.05 × 10^−7^	6.39 ± 2.85 × 10^−7^
	25239	2.13 ± 0.57 × 10^−2^	1.99 ± 3.45 × 10^−5^	0

^a^Results are given as the number of antibiotic-resistant transformants relative to the total number of cells (grown in standard media), with average ± standard deviation of three independent assays shown.

To determine whether secreted nuclease was sufficient to complement the competence defect of *ΔnucM* mutant strains, transformation assays were carried out with cells resuspended in cell filtrate and integrative plasmid pMComCm DNA. Results from assays with *ΔnucM* strains in filtrate from growth of wild type strains (containing secreted NucM) produced no transformants (Table 2). These data indicate that providing exogenous nuclease does not facilitate transformation of DNA and suggests that the nuclease needs to be associated with the cell to facilitate competence.

**Table 2:** Transformation efficiency of wild type and isogenic *ΔnucM* mutants with exogenous-complementation of NucM.

Strain	WT strain in *ΔnucM* filtrate (−secreted NucM)^a^	*ΔnucM* 1 strain in WT filtrate (−secreted NucM)^a^	*ΔnucM* 1 strain in WT filtrate (+secreted NucM)^a^
25328	1.74 ± 0.1 × 10^−2^	0	0
25239	4.12 ± 0.1 × 10^−2^	0	0

^a^Results are given as the number of antibiotic-resistant transformants relative to the total number of cells (grown in standard media), with average ± standard deviation of three independent assays shown.

### NucM contributes to cell-cell aggregation and biofilm formation

**Figure 4:**
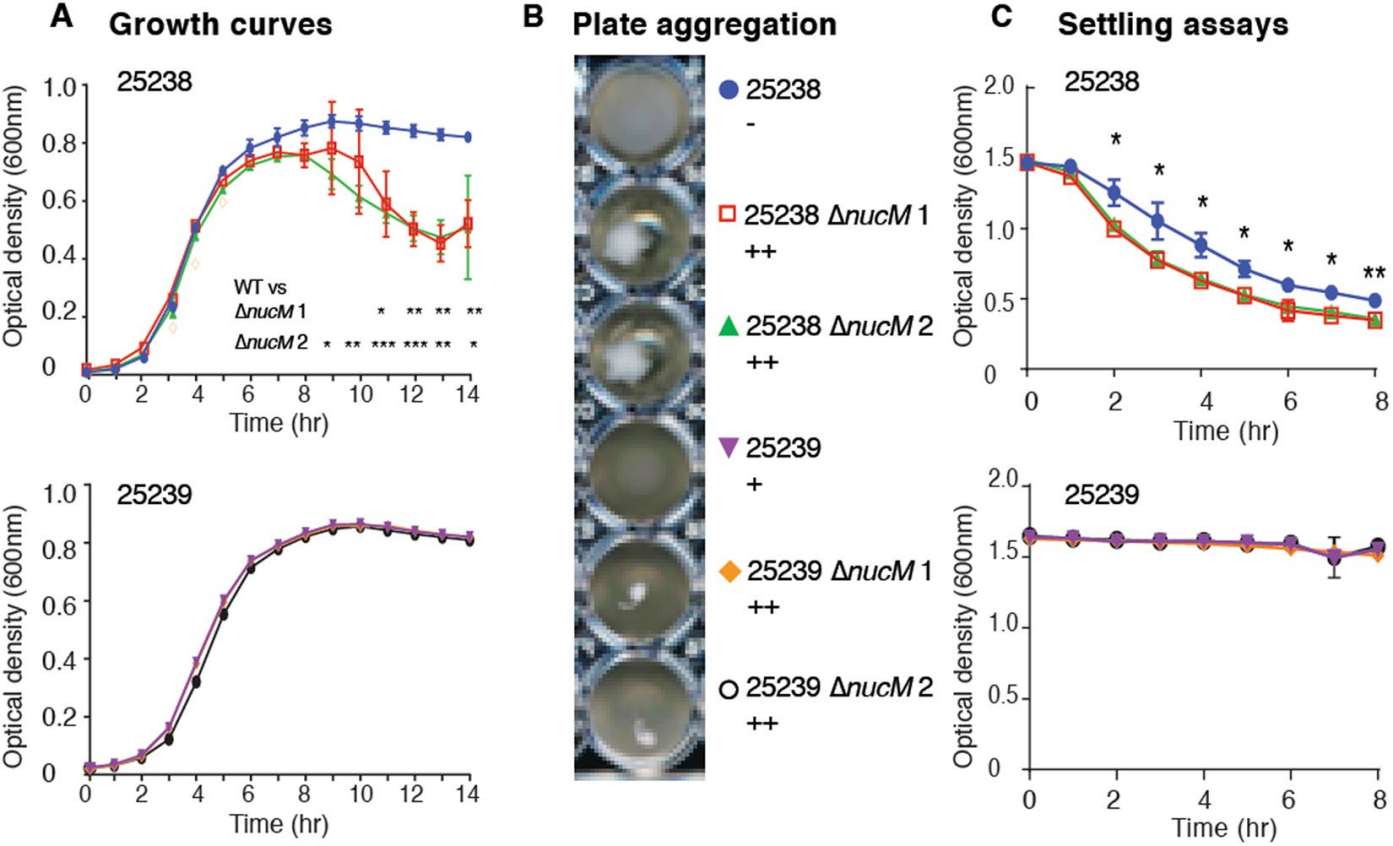
Cell-cell aggregation of *M*. *catarrhalis* wild type and isogenic *ΔnucM* mutants. (**A**) Growth curve of *M*. *catarrhalis* strains, grown overnight in CDM at 37 °C with aeration, using 0.5 mL aliquots in a 48-well plate format. Assays were carried out in triplicate and OD_600_ values were measured in a Tecan Infinite 200 Pro plate reader, with mean and standard deviation shown. Significance of mean differences was tested by t-test for unpaired samples not assuming equal standard deviations in PRISM; ***p < 0.0005; **p < 0.005, *p < 0.05. (**B**) Example wells from plate assay, showing aggregates in wells with *ΔnucM* strains. The level of aggregation seen for each strain is indicated: ‘−’ for no aggregation, ‘+’ for minor aggregation, ‘++’ for aggregation. (**C**) Settling curves shown for *M*. *catarrhalis* 25238 (top) and 25239 (bottom). Assays were carried out in triplicate with OD_600_ readings taken at the time points indicated. Average and standard deviations are shown. Significance was tested by t-test for unpaired samples not assuming equal standard deviations in PRISM; **p < 0.005, *p < 0.05.

Data from our degradation assays indicate that the majority of NucM is secreted from the cell, rather than membrane associated, suggesting that NucM may have other roles in addition to competence for *M*. *catarrhalis*. In chemically defined media, growth of 25238 *ΔnucM* mutants showed anomalous optical density readings at time points after 7 hours (Fig. 4A), and examination of plates after overnight growth showed formation of cell aggregates in wells (Fig. 4B). This suggested that *nucM* inhibited cell aggregation, and this was further investigated by settling assays, in which the optical densities of stationary cell suspensions were measured over time. The 25238 Δ*nucM* strains aggregated and settled faster than the parental strain (Fig. 4C), but 25239 parental and Δ*nucM* strains do not show settling over the duration of the assay (Fig. 4C) despite the aggregates seen in Fig. 4B. The lesser effect of *nucM* deletion in strain 25239 compared to 25238 may be due to the less robust biofilm formed by the wild type strain 25239 relative to 23258 (Fig. 4B). These data indicate that NucM mediates factors that affect cell-cell contact and aggregation of strains.

**Figure 5:**
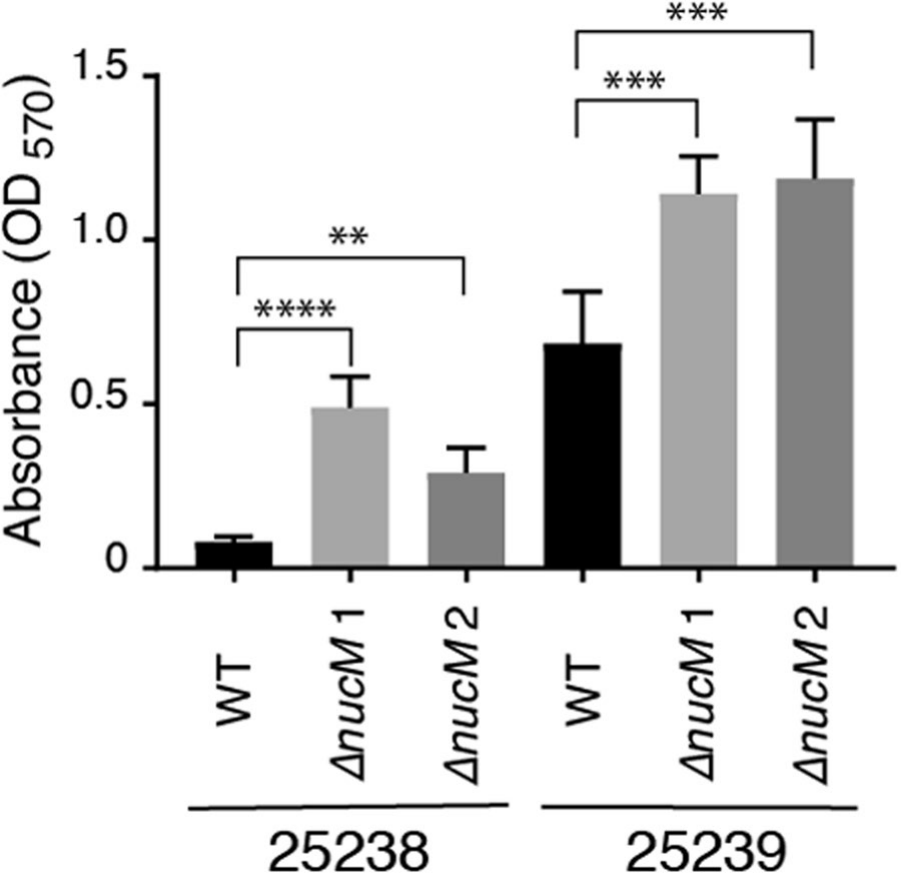
Static plate biofilm assay of *M*. *catarrhalis* wild type and isogenic *ΔnucM* mutants. Assays were carried out in 48-well plate format over 20 hours, and biofilm biomass quantified by crystal violet staining and absorbance at 570 nm. The data presented represent the average of six replicates, and the standard deviation is shown. Significance was tested by t-test for unpaired samples not assuming equal standard deviations in PRISM; ****p ≤ 0.0001, ***p ≤ 0.0005; **p ≤ 0.005.

**Figure 6:**
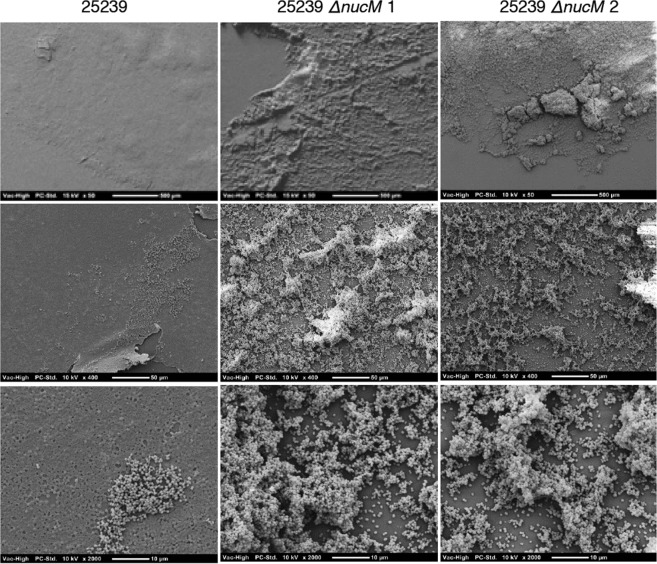
Scanning electron microscopy of static plate biofilm of wild type and isogenic *ΔnucM* mutants. Assays were carried out in 48-well plate format with coverslips over 20 hours, with biofilms washed, fixed and dehydrated in plates. Coverslips were removed, mounted and gold-sputtered for visualisation on SEM. Representative images of *M*. *catarrhalis* 25239 wild type and *ΔnucM* mutants are shown, taken at 50x, 400x and 2000x magnification (top, middle and bottom rows, respectively) and scale bars are indicated on each panel.

Given the role of the nuclease on cellular aggregation, it was hypothesised that microcolony formation and subsequently, biofilm formation might also be affected. Therefore, the effect of NucM on biofilm formation was investigated in two independent biofilm assays. For static plate assays, *M*. *catarrhalis* wild type and Δ*nucM* strains were incubated overnight in 48-well tissue culture plates, and biofilm biomass was quantified by crystal violet staining the following day. In these assays the *ΔnucM* mutants had higher biofilm biomass than parental wild type strains (3.6-fold to 6-fold higher for 25238 strains, and 1.7-fold higher for 25239 strains; Fig. 5). To visualise possible structural differences in biofilms formed, assays were repeated with coverslips for scanning electron microscopy. As seen with crystal violet assays, *M*. *catarrhalis* 25238 strains adhered poorly to the plastic, and cell aggregates were lost during processing. However, coverslips with adhered *M*. *catarrhalis* 25239 wild type and *ΔnucM* mutants showed significant differences in biofilm mass and morphology (Fig. 6). Wild type strains produced relatively flat films that coated the coverslips in an even layer of cells in a mat-like organisation. In contrast, *ΔnucM* mutants produced floating films in the assay, that when dehydrated and imaged showed more three-dimensional structure. Images show that the coverslips are not completely covered, however cells aggregate into structures that resemble mature biofilm architecture (Fig. 6).

**Figure 7:**
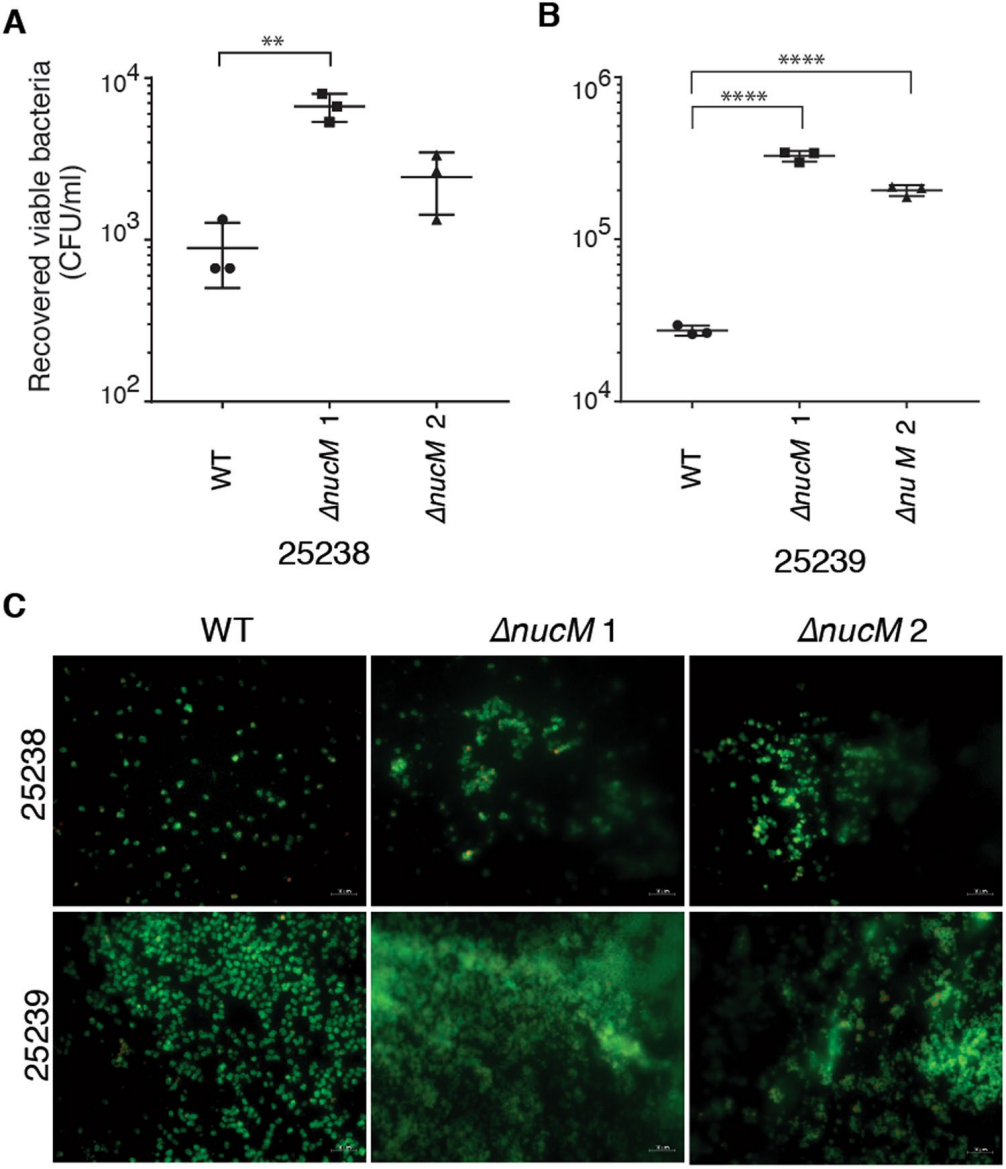
MBEC^TM^ biofilm assays of *M*. *catarrhalis* wild type and isogenic *ΔnucM* mutants. Assays were carried out in 96-well plates for 48 hours, at 37 °C with aeration. Viable colony forming units of biofilms attached to MBEC^TM^ pegs were assessed by sonication of pegs and enumeration on BHI agar for 25238 (**A**) and 25239 (**B**). Significance was tested by two-sided t-test for unpaired samples not assuming equal standard deviations in PRISM; ****p ≤ 0.0001, ***p ≤ 0.0005; **p ≤ 0.005. (**C**) Fluorescence live/dead staining with SYTO9 (green) and propidium iodide (red) of 25238 (top) and 25239 biofilms (bottom) on MBEC^TM^ pegs.

For MBEC^TM^ biofilm assays, 96-well plates were inoculated with suspensions of *M*. *catarrhalis* wild type and *ΔnucM* strains, and a lid with 96 identical pegs that protrude down into the wells was inserted into the cell suspension to scaffold biofilm growth. Biofilms were grown for 48 hours with aeration, and biofilm formation on pegs was assessed by fluorescence microscopy and enumeration of viable cells recovered from the established biofilms. This assay demonstrates that up to 1-log fewer viable cells were recovered from wild type 25238 and 25239 biofilms than for isogenic Δ*nucM* strains (Fig. 7A,B, respectively). Visualisation of biofilms with fluorescence microscopy revealed that wild type *M*. *catarrhalis* attached to MBEC^TM^ pegs individually or as diplococci. In contrast, *ΔnucM* strains show larger aggregations and clumps of cells attached to pegs, indicating increased cell-cell interactions. These data further confirm that deletion of *nucM* increases biomass and alters biofilm structure.

## Discussion

Extracellular nucleases are known to be involved in the virulence of a range of bacterial species, where they mediate phenotypes such as competence, biofilm formation and degradation of neutrophil extracellular traps. This study identifies and characterises the gene encoding the extracellular nuclease of *M*. *catarrhalis*, a member of the Nuc superfamily (also called DNA/RNA nonspecific endonucleases). This group is prototypically represented by the *Serratia* endonuclease^41,42^, and includes nucleases such as EndA from *S*. *pneumoniae*^12^, nuclease A from *Anabaena* spp^43^, and is characterised by a conserved DRGH motif at the catalytic core of these endonucleases present in residues 174–177 in NucM, and putative co-factor binding at residues 198 and 205. Other than these residues, however, *M*. *catarrhalis* NucM shares little sequence similarity with other members of the groups – for example having less than 12% sequence identity to EndA – so few inferences on function can be made based on previous studies.

This study has shown that NucM is an extracellular nuclease capable of digesting DNA and RNA in the external milieu and is non-essential to bacterial growth. NucM appears to be constitutively expressed under the conditions tested in this study, which correlates with genome-wide expression studies of *M*. *catarrhalis* BBH18^38^, although levels of expression vary under different nutritional conditions with differences seen in rich BHI broth compared to chemically defined broth. NucM is predominantly secreted, although a small portion of it remains membrane-associated and facilitates *M*. *catarrhalis* transformation.

Natural competence, the ability to take up and integrate DNA from the environment, is a widespread phenomenon amongst bacteria^20,48,49^. A general type IV pili mediated pathway for competence is seen in both Gram-positive and Gram-negative species (with the exception of *H*. *pylori* which uses a type IV secretion system-based pathway), and homologues of many of the major components have been identified in various species^20,49^. The exception to this is nucleases, which are generally unknown despite the general assumption that DNA entering the cell is single stranded^20^. The role of nucleases in competence is primarily modelled on EndA from *S*. *pneumoniae* and NucA from *Bacillus subtilis*. EndA was identified as an entry nuclease for DNA uptake over 40 years ago^50^, and is constitutively expressed^12,51^. EndA remains evenly membrane distributed until periods of cell competence, at which point it localises to mid-cell focal points in association with ComEA proteins and dsDNA^11^. Similarly, in the case of NucA from *B*. *subtilis*, ComEA proteins bind dsDNA and deliver it to membrane-localised NucA, which linearises the DNA for entry into the cell^52^. In this latter case, the authors suggest the role of NucA is to provide termini for DNA uptake initiation, as pre-digested DNA is taken up at the same rate in the *ΔnucA* mutant as undigested DNA by the wild type^52^. Interestingly, our studies show that NucM acts in a sequential manner with DNA being nicked then linearised before full degradation occurs, and this may suggest that NucM also catalyses termini formation for transformation efficiency in *M*. *catarrhalis*.

The role of nucleases in DNA uptake in Gram-negative pathogens is largely unstudied, although extracellular nucleases have been identified in naturally competent species, such as *N*. *gonorrhoeae*^18,30^ and *H*. *influenzae*^13^. In the case of *V*. *cholerae*, extracellular nucleases are detrimental to competence^53^. Therefore, to the best of our knowledge, the *M*. *catarrhalis* NucM protein characterised in this study is the first reported example of a ββα family entry-nuclease in a Gram-negative species. Further studies will be needed to elucidate its exact mechanism of action, especially as Gram negative bacterial species have outer and inner membranes that DNA must transfer across (processes known as DNA uptake and translocation, respectively^54^). For example, *M*. *catarrhalis* has putative annotated *comEA* and *comEC* reading frames (annotated as MC25239_RS08670 and MC25239_RS06360 in *M*. *catarrhalis* 25239, respectively), but whether they interact with NucM to facilitate DNA uptake is yet to be determined. In addition, *M*. *catarrhalis* competence appears to be a constitutively expressed phenotype^55^ as is also seen for the *Neisseria* species, but in contrast to regulated competence expression seen for most other bacterial species^48^. *M*. *catarrhalis* also shares other aspects of *Neisseria* competence, including the use of type IV pili subunits and *pilQ* secretin to pass the outer membrane^56,57^, but further similarities remain to be investigated, such as whether *M*. *catarrhalis* DNA uptake is facilitated by a DNA uptake sequence^54^. Regardless, NucM-facilitated competence is likely to enhance the fitness of *M*. *catarrhalis* as the transformed DNA may be used to repair genome damage, facilitate allelic shuffling or replacement, and/or allow acquisition of new genetic material^20,58^.

The genome maintenance and genome diversification that is facilitated by transformation can provide evolutionary advantages to the species, by enabling increased adaptability to stressful and/or rapidly changing environments^20,58^. These advantages may work in the short term, allowing the cell to repair damage caused by immune cell killing mechanisms, and to evade established immune responses by switching out alleles with targeted epitopes. In addition, the acquisition of new genetic material may lead to eventual diversification of the species into lineages with different pathogenic profiles. At present, the *M*. *catarrhalis* population is split into two main clades, with the RB1 lineage considered to be more closely associated with pathogenicity than the RB2/3 lineage^59^. The constitutive competence facilitated by NucM may eventually lead to further diversification, such as is seen with *Neisseria meningitidis*, for which the population is sub-divided into numerous clonal complexes, often with specific geographical, temporal or disease associations^60^.

Interestingly, data from this study indicate that only a small proportion of membrane-associated nuclease is needed for transformation, which is similar to what has been seen with the pneumococcal EndA^12^. It is unclear how widespread this phenomenon might be, as nuclease pleiotropy is otherwise largely unstudied, however for many other species, expression of the nuclease is controlled and may limit other functional activities (e.g., in *B*. *subtilis*, the competence associated nuclease is expressed with the competence operon^61^); or the role of the nuclease in competence has not been studied. Future studies may address this knowledge gap, and it may be of particular interest to examine the *N*. *gonorrhoeae* nuclease, as this species is also constitutively competent like *M*. *catarrhalis*. The role of the *N*. *gonorrhoeae* nuclease in competence is unclear, however studies indicate Nuc digests gonococcal genomic DNA more slowly than genomic DNA from other species (potentially due to methylation patterns^30^). Restriction–modification (R-M) systems are differentially distributed in the *M*. *catarrhalis* RB1 and RB2/3 lineages^62^ and it would be interesting to determine whether this links with DNA uptake specificity^54^ of this species.

Whilst the role of NucM in competence is important, it likely fulfils other functions for the physiology or pathogenesis of *M*. *catarrhalis*. Indeed, our data indicate that secreted NucM is involved in cell-cell interactions, and biofilm formation. *M*. *catarrhalis* is typically a highly aggregative species, and colonies will remain intact when pushed across the surface of agar^63^. For *M*. *catarrhalis* strain 25238, which shows aggregation typical of the species, *ΔnucM* mutants show increased aggregation in plates and settling assays compared to parental strains. Similar results were not seen for *M*. *catarrhalis* 25239, as this strain has atypically low aggregation, however this strain forms significantly more biofilm than strain 25238. In static biofilm assays, *ΔnucM* mutants for both strains showed increased biomass relative to parental strains, and the adherence of 25239 strains to coverslips allowed visualisation of bacterial community structure under SEM.

The link between biofilm and extracellular nucleases is extracellular DNA (eDNA) that acts as a scaffold in bacterial biofilms, as first demonstrated by *Pseudomonas aeruginosa* biofilm dissolution after treatment with DNaseI^26^. Interestingly, it appears that the source of the eDNA may not be important, with studies suggesting that exogenously added DNA^64^ or DNA and actin from lysed human neutrophils can enhance biofilm formation^65^. Nucleases have since been shown to disrupt eDNA scaffolding and cause biofilm formation defects in a range of pathogenic bacteria, and the potential of using nuclease enzymes as therapeutics or preventatives to prevent biofilm formation for a range of bacterial species has been considered^25^. In cases where the biofilm-forming bacteria produce an extracellular nuclease, disruption of the nuclease gene results in biofilm dysfunction, increasing biomass, but potentially limiting dispersal^13,14,16^, and often nuclease mutants have altered persistence or survival *in vivo*^10,13,17,35^. Our data indicate that NucM is needed to mediate cell-cell interactions, and in the absence of the nuclease, biofilm biomass increases, possibly due to an enhanced three dimensional biofilm architecture afforded by eDNA scaffolding that accumulates in the nuclease mutant, and cells may also be unable to escape the eDNA scaffold and disperse.

The effects of NucM on cell aggregation and biofilm formation have implications for the pathogenesis of *M*. *catarrhalis*. Our data suggest that NucM expression is decreased under early growth stage conditions with limited nutrients (i.e. chemically defined media compared to rich media), and this may correlate with early stages of infection *in vivo*, allowing *M*. *catarrhalis* cells to aggregate and establish biofilms. Increases in nuclease production due to nutritional influx, or accumulation of secreted nuclease may then trigger dispersal of *M*. *catarrhalis* to new sites. It is also interesting to consider how NucM may affect pathogens that co-infect with *M*. *catarrhalis*, such as the other otopathogens *S*. *pneumoniae* and *H*. *influenzae*. Co-infection of *M*. *catarrhalis* and other pathogens has been known to have synergistic effects – for example, *M*. *catarrhalis* can protect other species from beta-lactam antibiotics and immune responses^66–68^. Co-infection with *M*. *catarrhalis* also alters colonisation dynamics in mouse models, particularly with the pnuemococci^68,69^, and *M*. *catarrhalis* can form multispecies biofilms with both *H*. *influenzae*^70^ and *S*. *pneumoniae*^68^. In the latter case, it is unclear what effect the constitutively secreted NucM might have on biofilm scaffolding, especially as both *S*. *pneumoniae* and *H*. *influenzae* also encode extracellular nucleases^13^. Further work is needed to determine whether nuclease production from these three pathogens is co-ordinated during co-infection, if production affects strains synergistically or antagonistically, and what the consequences of this may be.

Collectively, the data presented in this study indicate NucM is an important protein for cell interactions and is likely to contribute to the growth and virulence of *M*. *catarrhalis* strains. NucM is shown to be highly conserved and catalyses the degradation of nucleic acids, and facilitates the competence of *M*. *catarrhalis* strains. NucM is also involved in cell aggregation and biofilm formation, and two strains of *M*. *catarrhalis* – 25238 and 25239, which have phenotypically different wild type aggregation levels – demonstrate that the loss of *nucM* results in higher levels of cell aggregation and biofilm formation. Taken together, this work suggests that NucM may be an important virulence factor for the persistence and pathogenesis of *M*. *catarrhalis* strains.

## Material and Methods

### Strains and plasmids

Bacterial strains and plasmids used in this study are listed in Table S1. *Escherichia coli* strains used for cloning were grown in Luria broth or on Luria agar plates (Oxoid, ThermoFisher Scientific), with antibiotics and supplements as needed [standard concentrations: ampicillin 100 µg/mL; kanamycin 50 µg/mL; chloramphenicol 10 µg/mL; X-gal 20 µg/mL]. *M*. *catarrhalis* strains were routinely grown in brain heart infusion (BHI) broth or on BHI agar (Oxoid). Where specified, chemically defined media (CDM) was used, containing per L: 5.6 g Na_2_HPO_4_, 2 g KH_2_PO_4_, 1 g NH_4_Cl, 0.1 g MgSO_4_, 10 mg CaCl_2_, 10 mL 60% sodium DL-lactate solution, 2.5 g L-aspartic acid (potassium salt), 1 g glycine, 0.5 g L-arginine monohydrochloride, 2 g L-proline, 0.1 g L-methionine, and 0.5 mL 0.1% freshly made Iron (II) sulphate heptahydrate (composition based on^71^. Antibiotics for *M*. *catarrhalis* were used at the following concentrations: kanamycin 20 µg/mL, chloramphenicol 1 µg/mL.

### Bioinformatics

Sequence data and automated annotations from genome sequencing were used to identify putative nuclease proteins^37,38^, which were characterised with BLAST^39^, Pfam^72^, Prosite^73^ and Phyre2^40^ for modelling, and PSORTb^74^ and SignalP^75^ for localisation. Additional *M*. *catarrhalis* nuclease homologues were identified by BLAST search in a custom *M*. *catarrhalis* genome database, compiled in Geneious (Biomatters Limited) from publicly available genome data. Homologues were extracted from BLAST results and compared using the Geneious software for amino acid differences, and similarity scores were calculated using the BLOSUM90 parameters with a threshold of 0. Additional homologues were identified by BLASTP at NCBI. Phylogenetic comparisons were made with MEGA7 software^76^, using the Maximum Likelihood analysis on CLUSTAL aligned protein sequences, with positions containing gaps or missing data eliminated.

### Nucleic acid degradation assays

Cell filtrates were typically prepared by growth of *M*. *catarrhalis* in media with aeration to mid- or late-log phase (unless otherwise specified), followed by centrifugation to remove whole cells and sterile filtration of the supernatant with a 0.22 µm syringe membrane filter to remove remaining cells. For free protein vs outer membrane vesicle (OMV) assays, filtrates were additionally ultracentrifuged at 100 000 × *g* for 1 hour, then supernatant removed and pellets washed with PBS three times. OMV pellets were resuspended in PBS for further analysis, and filtrates, supernatant and OMV preparations were plated on to media to confirm that no intact viable cells were present in these fractions. For degradation assays, 100 ng of DNA, or 200 ng of RNA, was co-incubated with cell filtrates overnight at 37 °C and visualised by agarose gel electrophoresis. For these assays, genomic DNA and RNA was extracted from *M*. *catarrhalis* strains 25328 and 25239, and non-specific plasmid DNA (pGEM, Promega) from *E*. *coli* DH5α.

### DNA manipulations

To make *ΔnucM* mutants, the *nucM* reading frame was replaced with a kanamycin resistance cassette by homologous recombination. To achieve this, a knock out cassette was constructed by flanking the pUC4K kanamycin resistance gene (amplified with nuc KO KF-nuc KO KR, see Table S2 for primers) with *M*. *catarrhalis* 25239 genomic DNA (gDNA) from upstream and downstream of the *nucM* gene (501 bp upstream and 523 bp downstream, amplified with primer pairs nucA KO LF-nucA KO LR and nucA KO RF-nucA KO RR, respectively). Fragments were assembled into the pGEM T-easy vector (Promega) with the NEBuilder kit (New England Biolabs) and transformed into *E*. *coli* DH5α for screening. Successful constructs were purified with the Wizard *Plus* SV minipreps DNA purification kit (Promega) and 1 µg of plasmid DNA was linearised with ScaI and transformed into *M*. *catarrhalis* strains by co-incubation on BHI agar at 37 °C 5% CO_2_ for 3 hours. Transformants were screened to confirm correct insertion using primer pairs nucA OUT F-ggt RO R and nucA OUT R-ggt LO F.

Complementation vector pMComCm was constructed by amplifying fragments of the *ggt* pseudogene (MC25239_RS02965) with primer pairs ggt LF F-ggt RF R and ggt RF F-ggt RF F. These flanks were combined by overlapping PCR and cloned into pGEM T-easy and sequenced. This construct was then digested with SmaI, and the chloramphenicol resistance cassette (*cat*) from *Campylocbacter coli*^77^ inserted after digestion with XbaI and BamHI, blunting with DNA polymerase I (Klenow fragment, NEB), and blunt end ligation. The resultant vector was screened to confirm successful cloning and determine cassette orientation and named pMComCm.

### Competence assays

For competence assays, overnight BHI agar cultures of *M*. *catarrhalis* cells were standardised to OD_600_ = 0.5, diluted to OD_600_ = 0.005 (approximately 5 × 10^4^ cfu/mL), and 100 µl aliquots were mixed with 100 ng of DNA and incubated for 3 hours at 37 °C in 5% CO_2_. Following incubation, cells were enumerated on plates with and without antibiotics, and transformation efficiency calculated as the number of antibiotic resistant transformants per total number of cells in the assays. Assays were carried out in biological triplicates. In cases where cell filtrates were used as exogenous NucM, filtrates from mid-log growth *M*. *catarrhalis* strains were prepared as described above, and used to resuspend cells to the required concentrations before assaying. For these assays, pWW115 plasmid DNA was extracted from *M*. *catarrhalis* 25238 and pMComCm plasmid DNA was extracted from *E*. *coli* DH5α. Genomic DNA (gDNA) and RNA was extracted from *M*. *catarrhalis* strains 25238 MComCm and 25239 MComCm.

### Growth curve and aggregation assays

*M*. *catarrhalis* strains were grown overnight on BHI agar, then subcultured into BHI broth the following day and grown to mid-log phase. Cells were then diluted to OD_600_ = 0.05 (or 0.1 for CDM) and 0.5 mL aliquots dispensed into 48-well tissue culture plates. Plates were grown with aeration overnight at 37 °C in a Tecan Infinite 200 Pro plate reader (Tecan, Switzerland) and read at 15 minute intervals for growth curve measurements (Tecan i-control software v 1.10.4.0). Assays were carried out in at least triplicate biological repeats.

For aggregation assays, cells were grown with aeration in BHI broth, and standardised to OD_600_ = 1.5. 2 mL of these suspensions were transferred to sterilised 8 mL cuvettes (Sarstedt), then cuvettes were sealed and incubated under stationary conditions. OD_600_ measurements were taken at periodic intervals above the level of cell sedimentation.

### Biofilm assays

For plate biofilm assays, *M*. *catarrhalis* strains were grown overnight on BHI agar and resuspended in BHI broth before standardisation to OD_600_ = 0.1 in CDM. Aliquots of 0.5 mL were dispensed into 48-well tissue culture plates, and plates grown stationary overnight at 37 °C, 5% CO_2_. The following day, media was aspirated from wells and cells stained with 0.05% crystal violet (Sigma-Aldrich) for 15 minutes. After staining, wells were washed three times with PBS and crystal violet was solubilised with 90% ethanol for quantification at 570 nm on the Tecan plate reader.

Peg biofilm assays were carried out using the MBEC^TM^ device (Innovotech, Inc; formerly the Calgary Biofilm Device) as described previously^78^. Briefly, cells were grown and resuspended in CDM with 10% BHI (for static plate assays for strain 25239), or BHI alone (for 25238 strains); and 150 µl of suspensions were used to inoculate flat-bottom 96-well plates. Lids with pegs were inserted into wells, and plates were incubated at 37 °C, 150 rpm, and 95% relative humidity for 48 hours. After incubation, the peg lid was removed and rinsed for 30 seconds in PBS (96-well plate, 200 µL in each well). The rinsed peg biofilms were transferred to a new 96-well plate containing fresh BHI recovery media. To assist the transfer of any remaining viable cells to the recovery media, the device was sonicated for 30 minutes (<20 °C), which sufficiently disrupts and dislodges the biofilms forcing them into the recovery media (BHI). The peg lid was then discarded, and biofilm CFUs were determined by serial dilution and plating of recovered biofilm cells on BHI agar plates.

### Scanning electron microscopy (SEM) and Fluorescence microscopy of biofilms

For SEM, static plate biofilm assays were carried out as described above, in the presence of 0.6 cm diameter hole-punched thermanox plastic coverslips (cell culture treated, ProSciTech) placed in the bottom of tissue culture plates. After incubation and aspiration of media, wells were washed three times with PBS, then fixed with aldehyde fixative [2% (v/v) gluteraldehyde; 5% (v/v) formaldehyde in PBS] for 30 minutes. Fixative was removed, and wells washed three times with PBS before two further washes with distilled water to remove salts. Samples were dehydrated by a series of five-minute incubations in increasing concentrations of ethanol in distilled water [30%, 50%, 75% and 90% ethanol, then two immersions in 100% ethanol], followed by 5 minutes with 50:50 ethanol:hexamethyldisilazane, then 5 minutes with 100% HMDS and air-drying. Samples were then mounted on 95 mm diameter studs (ProSciTech) with carbon tape and sputter-coated with gold using a JEOL NeoCoater MP-19020NCTR (JEOL). Samples were viewed using a JEOL NeoScope (JCM-5000), at 10 kV under high vacuum at the magnifications specified.

For visulaisation of biofilms formed on pegs, MBEC^TM^ biofilms were cultivated as detailed above, then rinsed for 30 second in saline (0.9%). After rinsing, pegs were incubated in 200 µl of 3.35 mM SYTO9 and 20 mM PI (LIVE/DEAD^TM^ *Bac*Light^TM^ Bacterial Viability kit L7007 (Life Technologies, Australia)) for 30 minutes at 30 °C in the dark. Stained biofilms on pegs were then rinsed for 30 seconds in saline (0.9%) and mounted using ProLong® Diamond Antifade Mountant (Thermo Fisher). Fluorescence microscopy was conducted on a Zeiss Axio Vert.A1 FL-LED equipped with filter sets, 38 (excitation: BP 470/40 nm, beamsplitter: FT 495 nm, emission: BP 525/50 nm) utilised for SYTO9, and 43 (excitation: BP 545/75 nm, beamsplitter: FT 570 nm, emission: BP 605/70 nm) applied for PI; and was carried out within an hour of staining and mounting.

## Supplementary information

Supplmentary
