# Supplementary material for: Moraxella catarrhalis NucM is an entry nuclease involved in extracellular DNA and RNA degradation, cell competence and biofilm scaffolding
Source: Sci Rep. 2019 Feb 22;9:2579. doi: 10.1038/s41598-019-39374-0 (PMC6384898; doi:10.1038/s41598-019-39374-0)
Supplement: Supplementary file 1 — Supplmentary [file 41598_2019_39374_MOESM1_ESM.pdf]

***Moraxella catarrhalis* NucM is an entry nuclease involved in extracellular DNA and RNA degradation, cell competence and biofilm scaffolding**

Aimee Tan[1], Wing-Sze Li[1], Anthony D. Verderosa[2], Luke V. Blakeway[1], Tsitsi Mubaiwa[1], Makrina Totsika[2] and Kate L. Seib[1*]

[1] Institute for Glycomics, Griffith University, Gold Coast, Queensland, 4215, Australia

[2] Institute of Health and Biomedical Innovation, School of Biomedical Sciences, Queensland University of Technology, Brisbane, Queensland, 4006, Australia

SUPPLEMENTARY INFORMATION

Table S1. Bacterial strains and plasmids used in this study

**Strains used in this study**		
**Strain**	**Features**	**Source/reference**
E. coli		
DH5α	Cloning strain	
M. catarrhalis		
25238	Wild type *M. catarrhalis* strain	ATCC
25238 *ΔnucM* 1	*nucM* knock out strain 1	This study
25238 *ΔnucM* 2	*nucM* knock out strain 2	This study
25238 MComCm	25238 *ggt::cat* (integrated *cat* gene in ggt complementation site)	This study
25239	Wild type *M. catarrhalis* strain	ATCC
25239 *ΔnucM* 1	*nucM* knock out strain 1	This study
25239 *ΔnucM* 2	*nucM* knock out strain 2	This study
25239 MComCm	25239 *ggt::cat* (integrated *cat* gene in ggt complementation site)	This study
**Plasmids used in this study**		
**Plasmid**	**Features**	**Source/reference**
pGEM T-easy	Commercial cloning vector	Promega
pUC4K	Commercial vector, kan^R	Amersham
pWW115	*M. catarrhalis* vector, $Spec^R$	E. Hansen (1)
pMComCm	*M. catarrhalis* integrative complementation vector, Cm^R	This study

(1) Wang, W. and Hansen, E.J. (2006) Plasmid pWW115, a cloning vector for use with *Moraxella catarrhalis*. *Plasmid*, **56**, 133-137.

Table S2. Oligos used in this study

**Oligo name**	**Sequence (5′-3′)**
nucA KO LF	gccgcgggaattcgattGACTGCTTGCCACTCATTGAC
nucA KO LR	caacgtggcGGTGCATCATAACTGTGAGCG
nucA KO KF	tgatgcaccGCCACGTTGTGTCTCAAAATCTC
nucA KO KR	tgaccaaatcTTAGAAAAACTCATCGAGCATCAAATGA
nucA KO RF	gtttttctaaGATTTGGTCATCAGTGCCATGC
nucA KO RR	ccgcgaattcactagtgatTGGTATGATAGATCGGCTTTGGT
nucA OUT F	CTTCTTAACACCCATCGCAACG
nucA OUT R	GCTCATCTTCCACTTACCAACAATG
ggt LF F	CGATACGGTGTATTGCCTTGG
ggt LF R	ctttctcgagtttgtacattagatctttcccgggCCTTCAGCATCATACCCCACC
ggt RF F	gaaagatctaatgtacaaactcgagaaaggcctCTGTACCCATTCGCCAGTTG
ggt RF R	GTGCACTGATGGCATCTTGG
ggt LO F	GTGGGTACGCCTGCTATCC
ggt RO R	GCGTGTTGCCATTGGACGG

* lowercase indicates oligo overhangs for cloning purposes

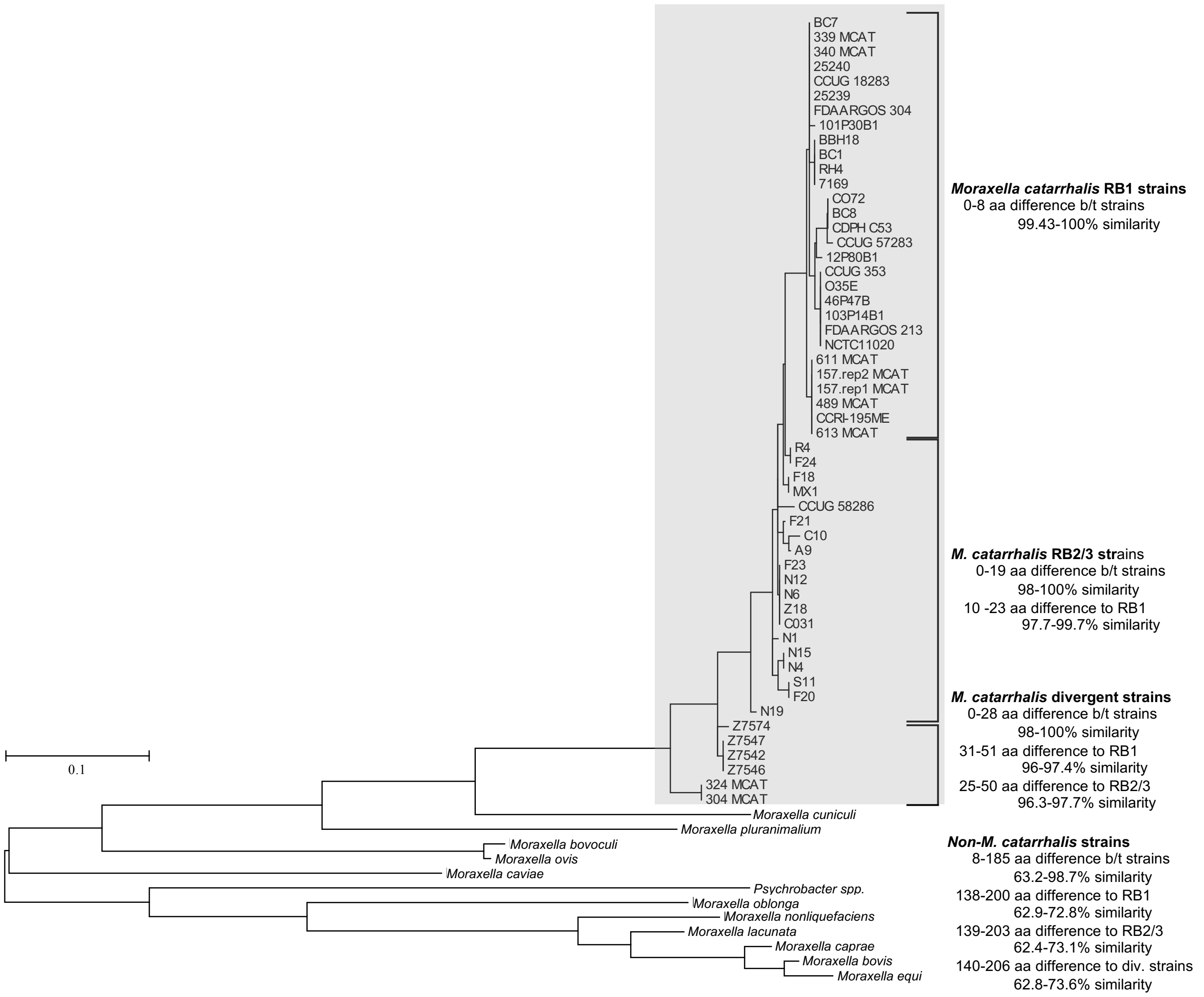

Fig. S1. Phylogenetic analysis of NucM in *M. catarrhalis* and related species. Maxmimum Likelihood phylogenetic tree of nuclease homologues in 54 strains of *M. catarrhalis* (boxed in grey) and 12 related species. Nuclease phylogeny reflects *M. catarrhalis* clades (as extrapolated from 2), indicated with brackets to the right of the tree. The range of amino acid (aa) differences and overall similarity percentage between (b/t) strains within clades and with respect to the RB1 clade are given to the right of the brackets.

(2) Earl, J.P., de Vries, S.P.W., Ahmed, A., Powell, E., Schultz, M.P., Hermans, P.W.M., Hill, D.J., Zhou, Z., Constantinidou, C.I., Hu, F.Z. *et al.* (2016) Comparative genomic analyses of the *Moraxella catarrhalis* serosensitive and seroresistant lineages demonstrate their independent evolution. *Genome Biol. Evol.*, **8**, 955-974.

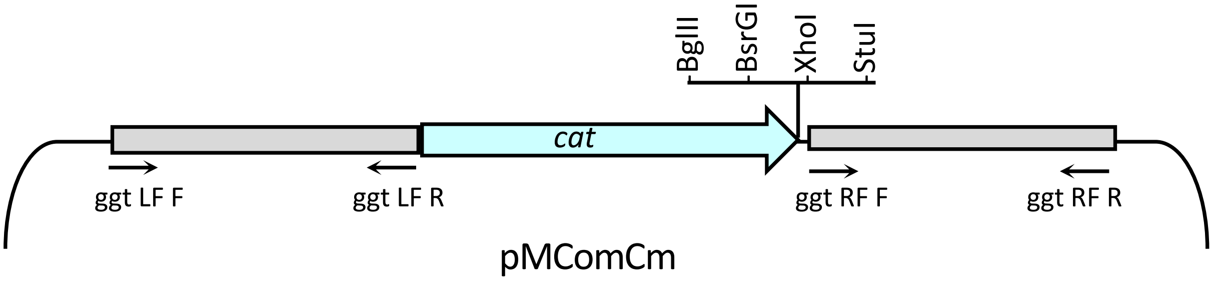

Fig. S2. Map of a fragment of the pMComCm complementation plasmid. The map shows the chloramphenicol resistance cassette (*cat,* large arrow), and multiple cloning site. Primers used to amplify flanks (grey rectangles) are shown as black arrows below (see Table S2 for primer sequences).

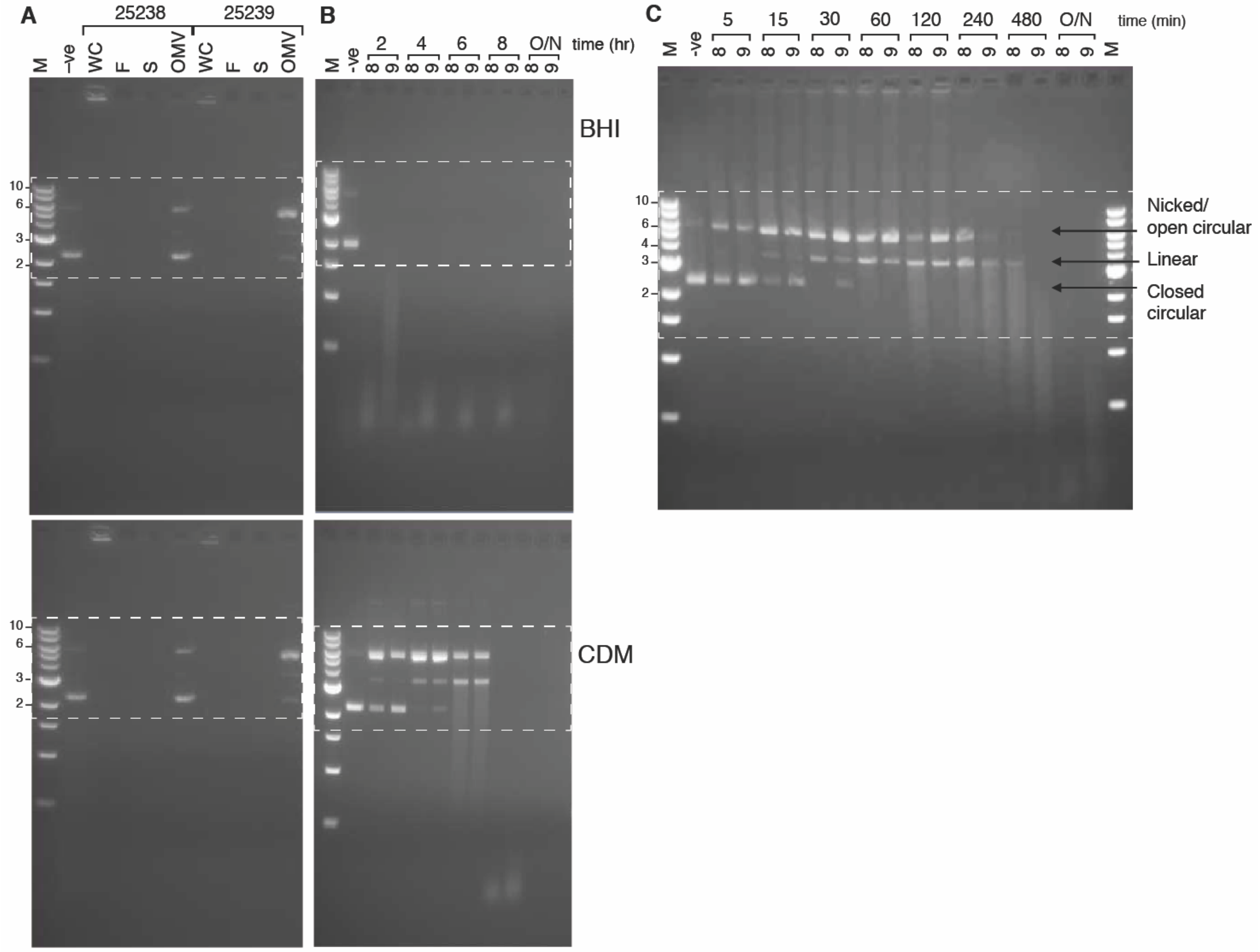

Fig. S3. Extracellular nuclease activity of *M. catarrhalis*. A. Nuclease activity in whole cell and secreted fractions of *M. catarrhalis* strain 25238 and 25239 cells grown in brain heart infusion media (BHI; top) and chemically defined media (CDM; bottom), co-incubated with plasmid DNA overnight. Fractions are whole cell (WC), cell free filtrate (F), supernatant from ultracentrifugation of OMVs (S) and outer membrane vesicles (OMV). Sizes are given for the marker (M; 1 kb ladder, NEB) in the bottom panel. **B.** Nuclease activity in BHI (top panel) and CDM (bottom panel) at 2 hour intervals (representing early-, mid-, late-log and stationary phases during aerobic growth) for *M. catarrhalis* strains 25238 (8) and 25239 (9), showing shift in plasmid forms from closed circular to nicked and linear forms in CDM isolated filtrate. **C.** Sequential degradation of plasmid DNA over time. Nuclease activity from cell-free filtrate from overnight aerobic growth of *M. catarrhalis* 25238 (8) and 25239 (9) strains in BHI, co-incubated with plasmid DNA for timed intervals of 5, 15, 30, 60, 120, 240 or 480 minutes, or overnight (O/N). Plasmid forms are indicated to the right of the gel, and sizes are given for the marker (1 kb ladder, NEB) to the left. Boxes with dashed lines indicate where the image was cropped for Fig.1.

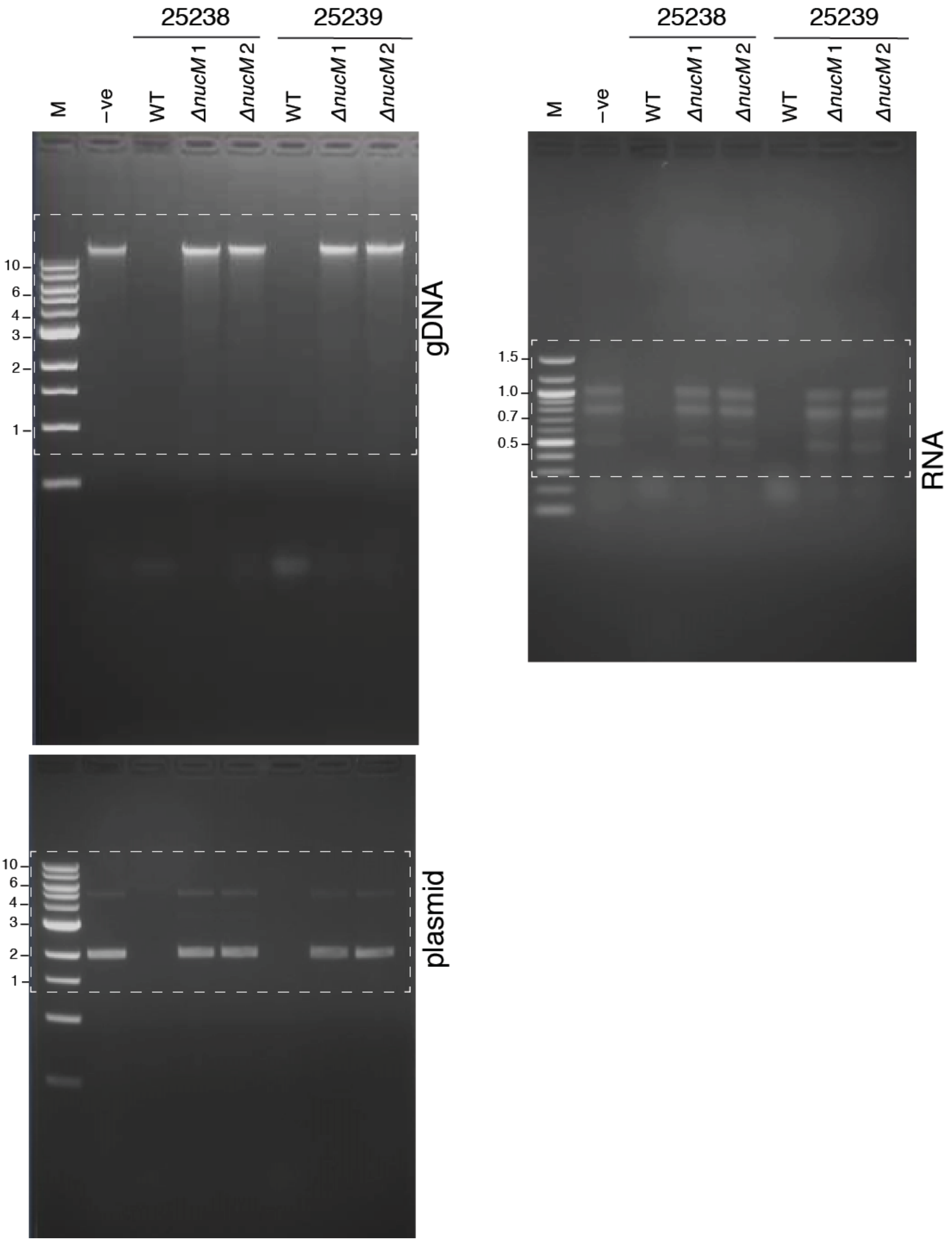

Fig. S4. Extracellular nuclease activity of *M. catarrhalis* wild type and isogenic *ΔnucM* mutants. Degradation assays of genomic DNA (gDNA), plasmid DNA or RNA incubated with filtered broth without cells (media only, -ve), or grown with wild type (WT) or isogenic *ΔnucM* knock out 25238 and 25239 strains. Boxes with dashed lines indicate where the image was cropped for Fig. 3.
